# The Behavioral and Emotional Profile of Pediatric Tourette Syndrome Based on CBCL in a Chinese Sample

**DOI:** 10.3389/fpsyt.2022.784753

**Published:** 2022-02-24

**Authors:** Yonghua Cui, Jiahui Chu, Yanlin Li, Ying Li

**Affiliations:** Department of Psychiatry, Beijing Children's Hospital, Capital Medical University, National Center for Children's Health, Beijing, China

**Keywords:** Tourette syndrome, CBCL, behavioral and emotional profile, ADHD, OCD, MDD

## Abstract

**Background:**

Tourette syndrome (TS) is a childhood-onset neuropsychiatric disorder that has a unique status of a quintessentially neuropsychiatric condition at the interface of neurology (movement disorder) and psychiatry (behavioral/emotional condition). However, the behavioral and emotional profile has seemed to be neglected in the literature thus far. This study aimed to investigate the behavioral and emotional profile of TS.

**Methods:**

A total of 124 patients aged 6–16 years with TS were included in this study, including age- and sex-matched health control, attention-deficit/hyperactivity disorder (ADHD), obsessive-compulsive disorder (OCD), and major depressive disorder (MDD) groups. The Child Behavior Checklist (CBCL) was used to screen the behavioral and emotional profile of the TS and other compared groups. The Yale Global Tic Severity Scale (YGTSS) was used to assess TS tic severity. Analysis of variance (ANOVA) was used to investigate the difference between the TS and other compared groups.

**Results:**

The results showed that the eight factors of the CBCL had no association with motor tics, vocal tics, or tic severity (*p* > 0.05). However, positive correlations were identified between functional impairments (subscales of YGTSS) and thought problems (TP) and rule-breaking behavior (RBB). Based on the eight-factor profile of the CBCL, TS showed a similar profile to MDD but different from ADHD and OCD, which showed similar profiles.

**Conclusions:**

Based on the assessment of the CBCL of TS, it was found that “pure” TS might show fewer behavioral and emotional problems than OCD, ADHD, and MDD. Similar behavioral and emotional profiles were identified between TS and MDD, but not OCD and ADHD. More attention needs to be paid to the thought problems and rule break problems in the CBCL in the screening stage, which might have a potential influence on the functional impairments of TS.

## Introduction

Tourette syndrome (TS) is a childhood-onset neuropsychiatric disorder characterized by multiple motor tics and one or more vocal tics that persist for at least 1 year (1). TS holds a unique status of a quintessentially neuropsychiatric condition at the interface of neurology (movement disorder) and psychiatry (behavioral/emotional condition) (2). It should be noted that TS presents with symptoms that seemingly mock the divisions between neurology (motor/vocal tic symptoms) and psychiatry/psychology (that is, motor, behavioral, and emotional symptoms) (3, 4). However, when investigating TS, we should focus not only on the movement dimensions (tic symptoms) of the condition but also on the behavioral and emotional symptoms of TS.

To the best of our knowledge, the behavioral and emotional symptoms of TS include attention problems, aggressive behavior, anxiety/depressive symptoms, obsessive-compulsive symptoms, and so on (5). Most of these symptoms are associated with the comorbidities of TS. For example, high rates of comorbid attention-deficit/hyperactivity disorder (ADHD) and obsessive-compulsive disorder (OCD) have been well-documented (6, 7). Moreover, major depressive disorder (MDD) has also been reported in TS (8, 9). It should be noted that comorbidities make the behavioral and emotional symptoms of TS more “complex.” Some studies have highlighted that “pure” TS (only tic symptoms) might be different from TS-Plus (that is, TS+OCD, TS+ADHD) (10, 11). TS+OCD has been regarded as one of the subtypes of TS, and TS+ADHD is another subtype (12). Some studies reported that the behavioral and emotional symptoms of TS were associated with OCD-related symptoms, while some reported OCD-related symptoms in TS (12–15). However, the behavioral and emotional profile of “pure” TS might need more evidence.

Furthermore, the comorbidities of TS, such as ADHD, OCD, and MDD, suggest that there is an overlap between TS and these mental disorders (16). Most studies focus on the differences between “pure” TS and TS plus other comorbid mental disorders, but few focus on the difference between “pure” TS and other “pure” mental disorders, especially at the behavioral and emotional levels.

The Child Behavior Checklist (CBCL) is one of the most important and stable tools for identifying the behavioral and emotional profiles of mental disorders (17, 18). It can be used to screen for TS, OCD, ADHD, MDD, and more (14, 19–24). Thus, the CBCL might be a good tool to present the differences in behavioral and emotional profiles among different mental disorders.

Therefore, this study aimed to investigate the behavioral and emotional profile of “pure” TS. Furthermore, we compared the differences in behavioral and emotional profiles between TS and other mental disorders (including OCD, ADHD, and MDD); the CBCL was used to present these differences. We hypothesize that TS may show different behavioral and emotional profiles when compared with OCD, ADHD, and MDD.

## Materials and Methods

### Participants

Children and adolescents (aged 6–16 years) with TS participated in this study. All participants were recruited from the Department of Psychiatry in Beijing Children's Hospital in China from 1 October 2019 to 1 September 2021. To identify patients with “pure” TS, the following criteria had to be met: (1) aged between 6 and 16 years, (2) met the Tourette syndrome diagnostic criteria according to the Diagnostic and Statistical Manual of Mental Disorders, Fifth Edition (DSM-5), (3) no central nervous system diseases or intellectual disability, and (4) no comorbidities of other mental disorders. Age- and sex-matched groups with MDD, OCD, and ADHD as well as healthy controls (HCs) were also recruited. To identify the patients with “pure” MDD, OCD, and ADHD, the criterion was that all patients in these groups should not have comorbidities with other mental disorders. For example, if the included patients belong to the MDD group, they should not have OCD, ADHD, TS, or other mental disorders. The HC group did not have any mental disorders.

This study was approved by the ethics committee of Beijing Children's Hospital of Capital Medical University, and written informed consent was obtained from the legal guardians of the participants or their parents.

### Scales for Assessments

#### YGTSS

The YGTSS is a semi-structured interview developed to assess the nature and severity of motor and vocal tics (25, 26). The assessment dimensions of the YGTSS include the number, frequency, intensity, complexity, and interference of vocal and motor tic symptoms, with a maximum score of 50 for tic severity (25 for motor and 25 for vocal tics) and 50 for the impairment caused by the tics, yielding a total maximum score of 100. The YGTSS is a widely used scale with excellent psychometric properties (27) and demonstrated excellent internal consistency (α = 0.91) in the present sample. A child psychiatrist was invited to perform the assessment of the YGTSS.

#### Children's Yale-Brown Obsessive Compulsive Scale (CY-BOCS)

The CY-BOCS is a semi-structured scale rated by a clinician. It was used to assess the severity of obsessive and compulsive behaviors during the previous week in patients with OCD aged 8–16 years (28). The obsessions and compulsion subtotals are derived by adding five items (time occupied, interference, distress, resistance, and degree of control, range: 0–4) related to obsessions (range: 0–20) and compulsions (range: 0–20), respectively. The total score is the sum of the obsessions and compulsion subtotals.

#### Depression Self Rating Scale for Children (DSRSC)

The DSRSC was used to assess depressive symptoms in young children aged 8–14 years. It measures the direction of disturbances felt in the past week (29). Three options include “Most of the time,” “Sometimes,” and “Never.” The scores for the scale are 2, 1, or 0, and the 18 item scores are then summed to give the total score. The maximum score is 36. The higher total scores are, the higher the depressive symptoms (30).

#### Swanson, Nolan, and Pelham Rating Scale–Fourth Version (SNAP-IV)

The SNAP-IV consists of 26 items rated on a 4-point scale (not at all, just a little, quite slightly, very much) (31). Three subscales were included (inattention, hyperactivity/impulsivity, and oppositional). The SNAP-IV was completed by parents and took ~15 min. Higher scores indicate more ADHD problem symptoms. Subscale scores are calculated by creating an average (32).

#### The Child Behavior Checklist (CBCL)

The Chinese version of the CBCL contains 118 specific behavioral and emotional problem items and two open-ended items (33). Each symptom question in the CBCL was scored 0 (not true, as far as you know), 1 (somewhat or sometimes true), and 2 (very true or often true). The CBCL contains eight factors: Anxious/Depressed (AD), Withdrawn/Depressed (WD), Somatic Complaints (SC), Social Problems (SP), Thought Problems (TP), Attention Problems (AP), Rule-Breaking Behavior (RBB), and Aggressive Behavior (AB). Liu et al. completed a regional survey in Shandong and reported that the two-week test-retest reliability was 0.90, and the internal consistency measured by Cronbach's α was 0.93 (34). Cronbach's α was also calculated in the present study and was 0.87 for the total scale. The CBCL was completed by parents or other caregivers. All CBCL assessments were performed using the QinChao Psychological Evaluation System (version 6.0) in the psychological assessment room in the Department of Psychiatry in Beijing Children's Hospital.

### Statistical Analysis

Statistical analyses were performed using the Statistical Package for the Social Sciences (SPSS Inc., Chicago, IL, USA, v25.0). First, we compared age using a *t*-test and the percentage of boys using a chi-square test. Second, the mean, standard deviation (SD), kurtosis, and skewness of the CBCL and its subscales were calculated for the TS group. Third, we calculated the Pearson correlation between the YGTSS and CBCL. Fourth, multivariate analysis of variance (MANOVA) was used to compare the CBCL and its subscales in different groups (TS, OCD, ADHD, MDD, and HC). MANOVA is a procedure for comparing multivariate sample means. As a multivariate procedure, it is used when there are two or more dependent variables and is often followed by significance tests involving individual dependent variables separately (35). Bonferroni correction was used when performing multiple comparisons among the different groups. To present the behavioral and emotional profiles, T-scores were used to calculate the eight factors based on CBCL. The T-score is one form of a standardized test statistic. Formulate T = (Z × 10) + 50, and formulate Z= (X–x)/SD. X is the value of one of the rough scores of the whole sample, and x is the mean of the whole sample. A radar chart based on T-scores was used to present the CBCL profiles of the different groups. The *p*-value ( ≤ 0.05) indicated significance against the null hypothesis.

## Results

### The Identification of the TS Group and Other Groups

**Figure 1:**
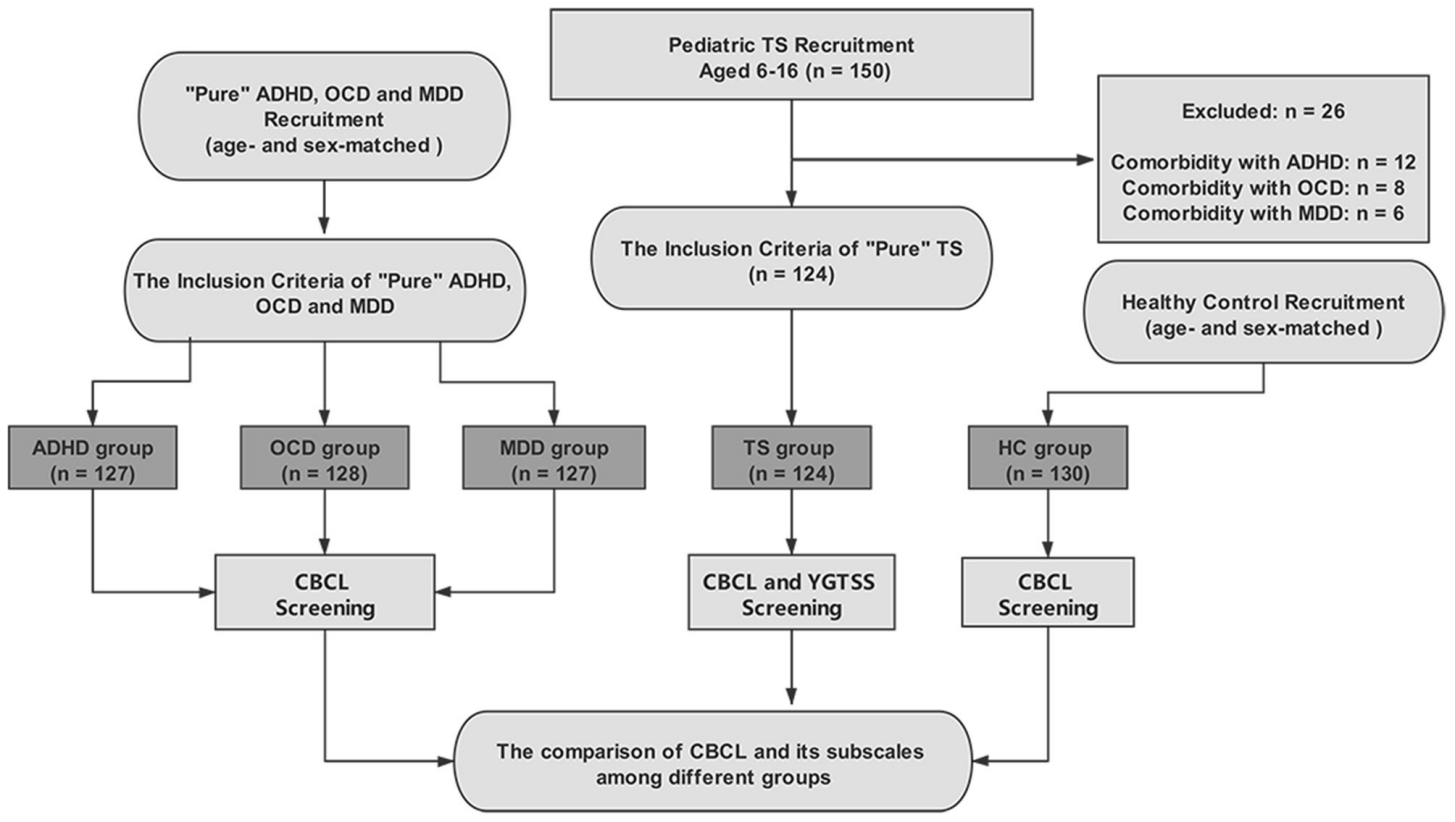
Flowchart of the selection criteria.

A total of 150 patients with TS were identified, but 26 were excluded due to comorbidities of other mental disorders. Finally, 124 patients with TS were included in the TS group. Furthermore, the age- and sex-matched groups included ADHD (*n* = 127), OCD (*n* =128), MDD (*n* = 127), and HC (*n* = 130) groups. For more details, see Figure 1.

The mean age of the patients in these groups was 10.37 ± 1.91 years (TS), 10.48 ± 2.74 years (ADHD), 10.59 ± 2.45 years (OCD), 10.48 ± 3.06 years (MDD), and 10.46 ± 2.38 years (HC). No significant age differences were identified in these groups (F = 0.128, *p* = 0.97). For the percentage of males, the TS group was 70.97%, ADHD group was 70.08%, OCD group was 70.31%, MDD group was 69.29%, and HC group was 68.46%; there was no significant difference among these groups (chi-square = 0.13, *p* = 1.00). The mean years of education of the patients in these groups were 4.36 ± 1.87 years (TS), 4.45 ± 2.25 years (ADHD), 4.58 ± 2.21 years (OCD), 4.48 ± 2.88 years (MDD), and 4.46 ± 2.19 years (HC). For the years of education, no significant age differences were identified in these groups (*p* > 0.05). The duration of illness (years) of the patients in these groups was 2.37 ± 1.89 years (TS), 2.48 ± 1.04 years (ADHD), 2.59 ± 1.45 years (OCD), 2.48 ± 1.06 years (MDD), and 2.46 ± 1.38 years (HC). For the duration of illness, no significant age differences were identified in these groups (*p* > 0.05).

We also calculated the YGTSS scores in the TS group, and the total YGTSS score was 21.63 ± 8.94 (motor tic: 12.77 ± 4.06; vocal tic: 6.24 ± 3.53; functional impairment 2.63 ± 4.88). The CY-BOCS score of the OCD group was 15.36 ± 4.45. Three subscales of SNAP-IV scores in the ADHD group were 1.75 ± 0.43 (inattention score), 1.83 ± 0.52 (hyperactivity/impulsivity score) and 0.83 ± 0.35 (oppositional score). The DSRSC score in the MDD group was 21.31 ± 5.83.

### The CBCL Profile of the TS Group

The mean, standard deviation, kurtosis, and skewness of the CBCL and its subscales were calculated in the healthy control (HC), TS, OCD, ADHD and MDD groups (for more details, see Table 1). Moreover, we calculated the Pearson correlation between the YGTSS and the CBCL. The results showed that the eight factors of CBCL had no association with motor tics, vocal tics, or tic severity (*p* > 0.05). However, positive correlations were identified between the function impairments (YGTSS) and the TP and RBB (subscales of CBCL) (for more details, see Table 2). In addition, we also calculated the mean, SD, range of scores of motor tics, vocal tics, and impairment of YGTSS in Supplementary Table 1.

**Table 1:** The descriptive statistic for the 8 factors of the CBCL in different groups.

	**HC (n** **=** **130)**		**TS (n** **=** **123)**		**OCD (n** **=** **128)**		**ADHD (n** **=** **127)**		**MDD (n** **=** **128)**	
**Syndromes**	**Mean (SD)**	**Skewness/ Kurtosis**	**Mean (SD)**	**Skewness/ Kurtosis**	**Mean (SD)**	**Skewness/ Kurtosis**	**Mean (SD)**	**Skewness/ Kurtosis**	**Mean (SD)**	**Skewness/ Kurtosis**
**A/D**	1.92 (1.85)	0.63/−0.62	4.27 (2.99)	0.14/−0.61	5.46 (3.75)	0.66/1.06	5.36 (3.67)	0.73/0.65	4.52 (3.03)	0.59/0.29
**W/D**	1.94 (1.75)	0.88/0.24	3.33 (2.08)	1.01/1.18	4.70 (2.97)	0.39/−0.48	4.28 (3.07)	1.16/1.46	3.84 (2.66)	4.81/−0.53
**SC**	2.18 (2.34)	1.72/4.75	4.76 (3.33)	0.99/0.15	4.65 (3.31)	0.56/−0.51	5.05 (3.77)	0.62/−0.16	5.28 (3.08)	0.18/−0.77
**SP**	2.35 (1.92)	0.56/−0.27	4.91 (2.67)	0.35/−0.66	5.38 (3.02)	0.45/0.05	5.74 (3.32)	0.49/0.80	5.16 (2.71)	0.25/−0.24
**TP**	1.68 (1.84)	1.29/1.43	3.94 (2.68)	0.56/−0.56	5.10 (3.40)	0.53/−0.26	5.15 (4.02)	1.80/4.78	3.74 (2.68)	0.90/0.46
**AP**	3.05 (2.25)	0.13/−1.05	4.96 (2.24)	0.71/−0.42	5.96 (2.87)	−0.01/−0.46	6.30 (3.11)	−0.02/0.27	5.15 (2.71)	0.19/−0.15
**RBB**	1.93 (1.80)	0.53/−0.88	3.37 (1.72)	0.86/2.42	4.21 (2.60)	0.85/0.48	4.33 (3.41)	1.60/4.30	3.70 (2.52)	0.64/0.25
**AB**	4.74 (3.62)	0.44/−0.79	6.69 (3.9)	0.61/0.26	9.67 (4.93)	0.76/0.40	10.31 (6.28)	0.64/0.48	8.93 (4.58)	0.43/−0.21
**Total problems**	23.76 (14.68)	−0.19/−1.50	42.79 (12.97)	0.18/0.18	52.29 (19.94)	0.58/1.00	54.90 (17.94)	1.46/4.69	47.48 (16.63)	0.01/−0.34

*A/D, Anxious/Depressed; W/D, Withdrawn/Depressed; SC, Somatic Complaints; SP, Social Problems; TP, Thought Problems; AP, Attention Problems; RBB, Rule-Breaking Behavior; AB, Aggressive Behavior; HC, Health Control; TS, Tourette Syndrome; OCD, Obsessive Compulsive Disorder; ADHD, Attention Deficit and Hyperactivity Disorder; MDD, Major Depressive Disorder; SD, Standard Deviation*.

**Table 2:** The Pearson correlation of YGTSS and the CBCL in Tourette syndrome (*n* = 124).

	**Motor Tic**	**Vocal Tic**	**Severity**	**Impairment**	**Total YGTSS**
A/D	−0.04	0.16	0.06	0.06	0.07
W/D	−0.11	0.07	−0.04	0.04	0.01
SC	0.04	0.12	0.10	0.10	0.13
SP	0.02	0.07	0.06	0.09	0.10
TP	0.04	0.14	0.12	0.18^*^	0.20^*^
AP	−0.02	0.16	0.08	0.08	0.10
RBB	−0.01	0.07	0.04	0.20^*^	0.17
AB	0.02	−0.01	0.01	0.11	0.09
Total CBCL	−0.00	0.15	0.09	0.20^*^	0.19^*^

*A/D, Anxious/Depressed; W/D, Withdrawn/Depressed; SC, Somatic Complaints; SP, Social Problems; TP, Thought Problems; AP, Attention Problems; RBB, Rule-Breaking Behavior; AB, Aggressive Behavior; CBCL, the Child Behavior Checklist; YGTSS, Yale Global Tic Severity Scale*;

* *p < 0.05*.

### Comparisons of CBCL Profiles Between the TS Group and Other Groups

**Figure 2:**
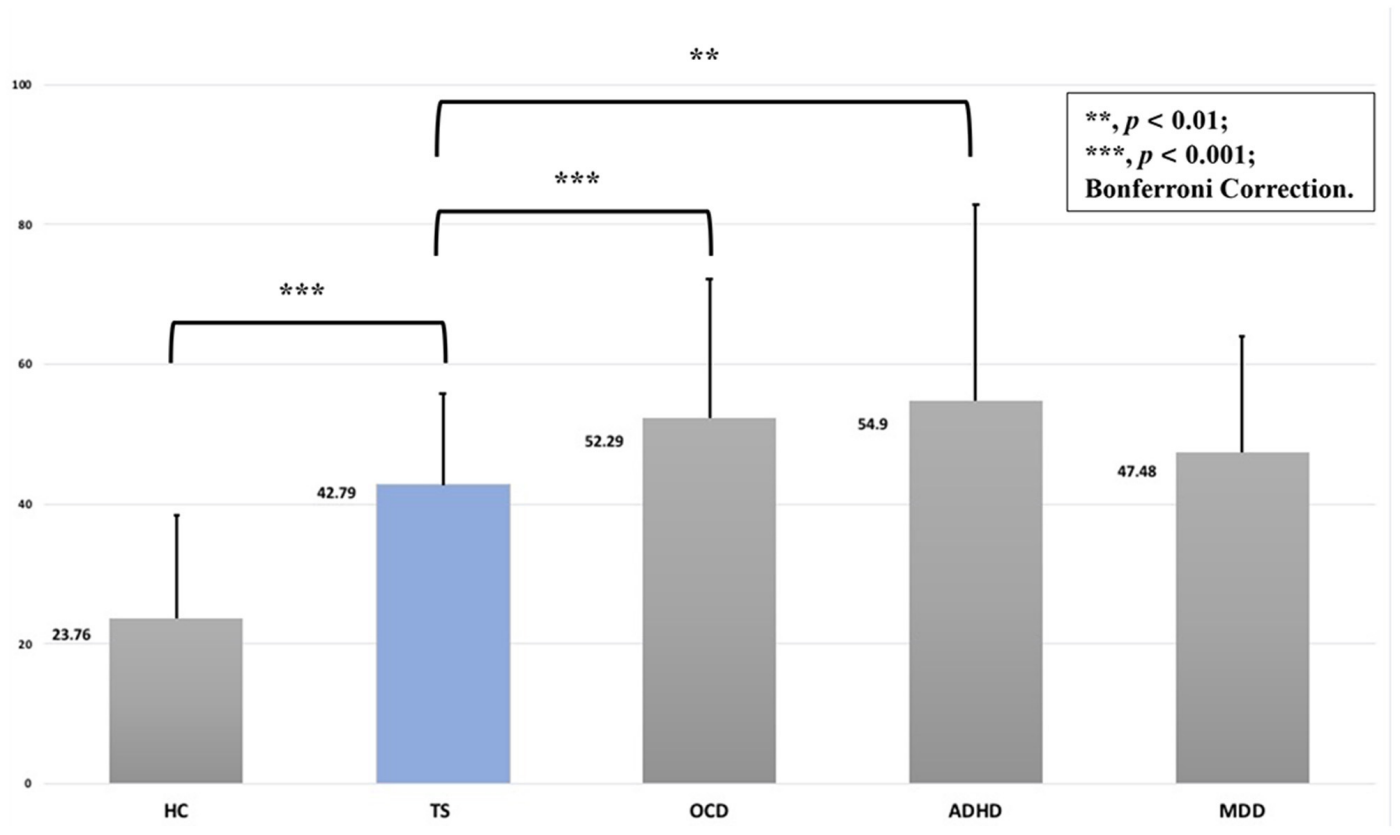
The CBCL total scores of the TS and other compared groups.

First, we compared the total CBCL scores of all the groups and found that F was 53.55 (*p* < 0.001) (for more details, see Supplementary Table 1). The *post hoc* test (Bonferroni correction) showed the following relationships with respect to CBCL profiles between the groups: TS > HC (*p* < 0.001), TS < OCD (*p* = 0.001) and ADHD (*p* < 0.001). No significant differences were identified between the TS and MDD groups (*p* = 0.530). For more details, see Figure 2.

Second, multivariate analysis of variance was used to compare the eight factors of the CBCL subscales in different groups (TS, OCD, ADHD, MDD, and HC). For the SC factor, TS > HC (*p* < 0.001), but no significant difference was identified among the TS, ADHD, OCD, and MDD groups. The SP factor showed the same pattern as SC, TS > HC (*p* < 0.001), but no significant differences were identified among the TS, ADHD, OCD, and MDD groups. For the A/D factor, TS > HC (*p* < 0.001), TS < OCD (*p* = 0.003) and ADHD (*p* = 0.006). The W/D factor showed the same pattern as A/D, TS > HC (*p* < 0.001), TS < OCD (*p* < 0.001) and ADHD (*p* = 0.004). The TP factor also showed the same pattern as A/D and W/D, TS > HC (*p* < 0.001), TS < OCD (*p* = 0.024) and ADHD (*p* = 0.016). The AP factor also showed the same pattern as A/D, W/D, and TP, TS > HC (*p* < 0.001), TS < OCD (*p* = 0.029) and ADHD (*p* = 0.001). For the RBB factor, TS > HC (*p* < 0.001) and TS < ADHD (*p* = 0.023). For the AB factor, TS > HC (*p* = 0.012), and TS < OCD (*p* < 0.001), ADHD (*p* < 0.001), and MDD (*p* = 0.002). For more details, see Tables 3, 4.

**Table 3:** MANOVA analysis based on CBCL.

**Total of CBCL and 8 subscales**		**Sum of Squares**	**df**	**Mean Square**	**F**	***P***
Total	Between Groups	78839.57	4	19709.89	53.55	*P* <0.01^**^
	Within Groups	232256.05	631	368.08		
	Total	311095.63	635			
A/D	Between Groups	1056.79	4	264.20	26.99	*P* <0.01^**^
	Within Groups	6176.45	631	9.79		
	Total	7233.24	635			
W/D	Between Groups	586.21	4	146.55	22.36	*P* <0.01^**^
	Within Groups	4134.87	631	6.55		
	Total	4721.07	635			
SC	Between Groups	818.04	4	204.51	20.05	*P* <0.01^**^
	Within Groups	6436.39	631	10.20		
	Total	7254.43	635			
SP	Between Groups	949.45	4	237.36	31.06	*P* <0.01^**^
	Within Groups	4822.98	631	7.64		
	Total	5772.43	635			
TP	Between Groups	1028.88	4	257.22	28.28	*P* <0.01^**^
	Within Groups	5739.36	631	9.10		
	Total	6768.24	635			
AP	Between Groups	824.19	4	206.05	29.20	*P* <0.01^**^
	Within Groups	4453.03	631	7.06		
	Total	5277.23	635			
RBB	Between Groups	479.95	4	119.99	19.38	*P* <0.01^**^
	Within Groups	3907.05	631	6.19		
	Total	4386.99	635			
AB	Between Groups	2735.38	4	683.86	30.24	*P* <0.01^**^
	Within Groups	14268.98	631	22.61		
	Total	17004.36	635			

*AD, Anxious/Depressed; W/D, Withdrawn/Depressed; SC, Somatic Complaints; SP, Social Problems; TP, Thought Problems; AP, Attention Problems; RBB, Rule-Breaking Behavior; AB, Aggressive Behavior; CBCL, the Child Behavior Checklist*;

** *p < 0.01*.

*Within group variation measures how much the individuals vary from their group mean, while Between group variation measures how much the group means vary from the overall mean*.

**Table 4:** *Post hoc* Tests (multiple comparisons, Bonferroni correction).

**Total and Subscales (CBCL)**	**TS vs. Groups**	**MD**	**SE**	**P**	**95% CI Lower Bound**	**95% CI Upper Bound**
Total	HC	19.03^**^	2.41	*P < * 0.01	12.23	25.83
	OCD	−9.50^**^	2.42	*P < * 0.01	−16.32	−2.68
	ADHD	−12.11^**^	2.43	*P < * 0.01	−18.95	−5.27
	MDD	−4.70	2.42	0.53	−11.52	2.13
A/D	HC	2.35^**^	0.39	*P < * 0.01	1.24	3.45
	OCD	−1.20^*^	0.40	0.03	−2.31	−0.08
	ADHD	−1.09	0.40	0.06	−2.21	0.02
	MDD	−0.26	0.40	1	−1.37	0.86
W/D	HC	1.40^**^	0.32	*P < * 0.01	0.49	2.3
	OCD	−1.36^**^	0.32	*P < * 0.01	−2.27	−0.45
	ADHD	−0.94^*^	0.32	0.04	−1.85	−0.03
	MDD	−0.50	0.32	1	−1.41	0.41
SC	HC	2.58^**^	0.40	*P < * 0.01	1.45	3.71
	OCD	0.11	0.40	1	−1.03	1.24
	ADHD	−0.29	0.40	1	−1.43	0.85
	MDD	−0.53	0.40	1	−1.66	0.61
SP	HC	2.56^**^	0.35	*P < * 0.01	1.58	3.54
	OCD	−0.47	0.35	1	−1.46	0.51
	ADHD	−0.83	0.35	0.18	−1.81	0.16
	MDD	−0.25	0.35	1	−1.23	0.74
TP	HC	2.27^**^	0.38	*P < * 0.01	1.20	3.33
	OCD	−1.16^*^	0.38	0.02	−2.23	−0.09
	ADHD	−1.21^*^	0.38	0.02	−2.28	−0.13
	MDD	0.20	0.38	1	−0.87	1.27
AP	HC	1.91^**^	0.33	*P < * 0.01	0.96	2.85
	OCD	−1.00^*^	0.34	0.03	−1.95	−0.06
	ADHD	−1.34^**^	0.34	*P < * 0.01	−2.29	−0.39
	MDD	−0.19	0.34	1	−1.13	0.76
RBB	HC	1.43^**^	0.31	*P < * 0.01	0.55	2.32
	OCD	−0.85	0.31	0.07	−1.73	0.04
	ADHD	−0.97^*^	0.32	0.02	−1.85	−0.08
	MDD	−0.34	0.31	1	−1.22	0.55
AB	HC	1.95^**^	0.60	*P < * 0.01	0.27	3.64
	OCD	−2.98^**^	0.60	*P < * 0.01	−4.67	−1.29
	ADHD	−3.62^**^	0.60	*P < * 0.01	−5.31	−1.92
	MDD	−2.24^**^	0.60	*P < * 0.01	−3.93	−0.55

*AD, Anxious/Depressed; W/D, Withdrawn/Depressed; SC, Somatic Complaints; SP, Social Problems; TP, Thought Problems; AP, Attention Problems; RBB, Rule-Breaking Behavior; AB, Aggressive Behavior; CBCL, the Child Behavior Checklist; HC, Health Control; TS, Tourette Syndrome; ADHD, Attention-Deficit/Hyperactivity Disorder; OCD, Obsessive-Compulsive Disorder; MDD, Major Depressive Disorder*;

* *p < 0.05*;

** *p < 0.01*.

**Figure 3:**
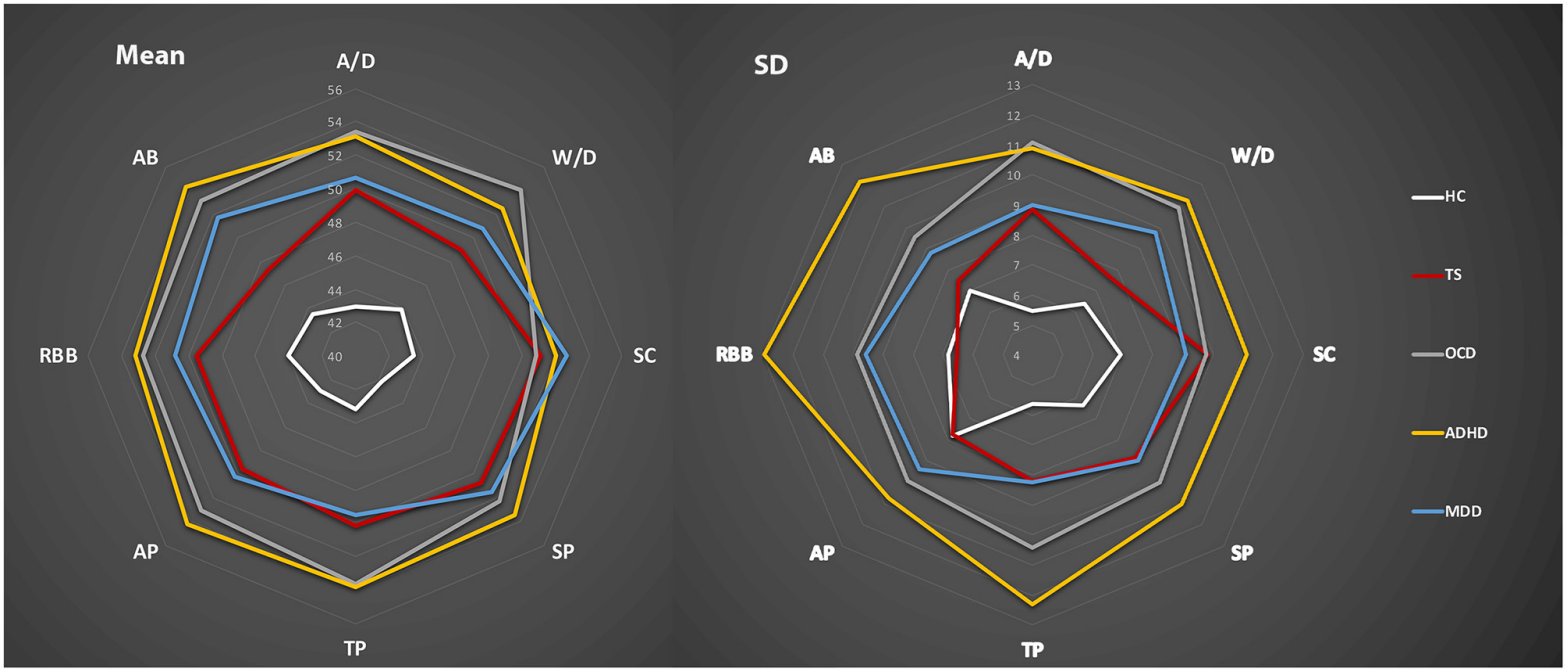
The CBCL profile of the TS and other compared groups.

Finally, we calculated the T-scores of each group based on the 8 subscales of the CBCL. The radar chart was used to present CBCL profiles of the different groups based on the mean and SD of 8 subscales based on the T-scores (see Figure 3). This suggested that, based on the eight-factor profile of the CBCL, TS showed a similar pattern to MDD but different from ADHD and OCD, which showed similar profiles. AB and W/D might be more “suitable” factors to present the difference among these groups rather than SC and SP.

In addition, considering the age effect for the CBCL of different groups, we divided the whole sample into a Young Group (6–11 years old) and an Old Group (12–16 years old). We also used the radar chart to present the CBCL profiles of these two groups based on the T-scores. There might be a higher score on the 8 subscales of the CBCL in the Old Group than in the Young Group. For more details, see Supplementary Figure 1.

## Discussion

This study aimed to compare the behavioral and emotional profile of “pure” TS with other mental disorders. The results showed that there was no correlation between tic symptoms and behavioral and emotional problems in “pure” TS. However, TP and RBB might have a potential influence on TS function. The “pure TS” group showed higher behavioral and emotional problems than the HC group and the same level of severity of behavioral and emotional problems as the MDD group. The “pure” OCD and ADHD groups showed higher-level severities of behavioral and emotional problems than the TS group. Moreover, the difference between the TS and OCD groups was mainly in the dimensions of A/D, W/D, TP, AP, and AB. The difference between the TS and ADHD groups was mainly in the dimensions of A/D, W/D, TP, AP, RBB, and AB. The difference between the TS and MDD groups was mainly in the AB dimension. TS, OCD, ADHD, and MDD showed the same levels of SC and SP. These results indicate that the “pure” TS might have a similar behavioral and emotional profile to “pure MDD” but a different profile compared to “pure” OCD and ADHD.

In the present study, it was found that the TS group might show a similar behavioral and emotional profile to the MDD group at the behavioral level. Rizzo et al. (36) reported that depression is significantly associated with TS factors, such as tic severity, but not obsessive compulsiveness. Furthermore, we found that there was no association between tic symptoms and behavioral/emotional problems. This implies that the “pure” tic symptoms and behavioral/emotional problems are two distinct cluster symptoms. We also identified higher CBCL scores in the TS group than in the HC group. This suggests that even the “pure” TS might have some behavioral and emotional problems different from tic symptoms. Indeed, Rizzo et al. (12) also reported that emotional lability represents an intrinsic core feature of Tourette syndrome that is unrelated to comorbidity. This implies that some emotional problems might also be associated with “pure” TS. This might be the most likely reason for the similar behavioral and emotional profile for “pure” TS and MDD. Further evidence is needed to investigate the association between tic symptoms and depressive symptoms in the future.

Furthermore, in the present study, we also found a difference in AB between “pure” TS and MDD. Compared with MDD, OCD, and ADHD, “pure” TS showed less aggressive problems. Aggressive behavior can be found in young patients with MDD (37), ADHD (38, 39), and OCD (40). Recently, a study reported that there was no association between aggressive behavior and tic symptoms, but comorbid ADHD and OCD increased the risk of aggressive behavior in patients with tic disorders (41). This suggests that aggressive behavior might be associated with comorbidities of TS but not with tic symptoms. This might be regarded as one of the most important behavioral indicators to distinguish the “pure” TS from OCD, ADHD, and MDD.

In the present study, we found confirmed differences between TS and ADHD at the behavioral level. “Pure” ADHD might present more ADHD-related behavioral problems (such as AP, AB, and RBB), which is different from tic symptoms. Furthermore, ADHD-related behavioral problems might also lead to emotional problems, which might be the reason why ADHD showed higher scores for A/D and W/D than TS. Furthermore, similar results were obtained when TS was compared with OCD. The OCD group showed higher levels of behavioral problems in AP, AB, RBB, and TP, as well as emotional problems in A/D and W/D. However, for the RBB, the TS and OCD groups showed similar scores. It should be noted that ADHD and OCD were the two most common comorbidities for TS, and both tend to persist (15). Both the genetic and phenotypic overlap of ADHD/OCD and TS have been reported (42). Moreover, it has been suggested that OCD and ADHD in TS predict worse outcomes of TS (43). The results of this study indicate that the comorbidities of ADHD and OCD in TS might increase behavioral and emotional problems and make the profile of TS more complex. Taken together, “pure” TS showed fewer behavioral and emotional problems, but with the comorbidities of ADHD or OCD, more behavioral and emotional problems might be identified. The dimension of OCD-related symptoms indicated that compulsivity is a clearly distinguished dimension for TS. How tics with both compulsivity and impulsivity, such as self-injurious behaviors and coprolalia, relate to the profile of CBCL in terms of the relationship between tics and OCD might be an important research direction for TS. Other behavioral and emotional problems, such as ADHD-related symptoms, might be another dimension of TS.

Notably, we identified the relationship between TP and the function of TS, which indicates that this dimension of behavioral and emotional problems might influence the functional impairment of TS. Although TP is clinically useful for identifying psychotic symptoms in children, it also includes items for the assessment of obsessive thoughts and compulsions, self-harm, picking at parts of the body, and more (44). These items have shown a robust association with the functional impairment of TS (45–47).

RBB has been shown to be associated with antisocial behavior problems, which are key factors in the development of youth violence and aggression (48, 49). This suggests that more attention should be given to RBB problems at the screening stage of TS. It should be noted that AB and RBB always showed a closed relationship. Therefore, there might be somewhat contradictory evidence that RRB had a significant correlation with functional impairments, while AB had a fairly low correlation. RBB had a much higher kurtosis than AB, and the correlation might be caused by the presence of a small number of TS participants with high RBB.

Compared to RBB, TP had less significant kurtosis. Therefore, TP may be more closely related to TS than RBB, and compulsivity indicated by TP may be a feature of TS, even if OCD is not comorbid.

Overall, in the present study, we found that “pure” TS might show fewer behavioral and emotional problems than OCD and ADHD. Similar behavioral and emotional profiles were identified between TS and MDD, but not OCD and ADHD. These results indicate that comorbidities (such as OCD and ADHD) might make the behavioral and emotional profiles more complex. Aggressive problems might be an important factor in distinguishing “pure” TS from OCD, ADHD, and MDD. Furthermore, we need to pay more attention to TP and RBB problems in the screening stage of TS, which might have a potential influence on the functional impairments of TS.

This study has two limitations. First, a limited number of participants were included in this study. A larger sample size and follow-up studies of behavioral and emotional profiles for TS are needed to confirm these results. Second, anxiety disorders are also a common comorbidity of TS but were not included in this study. Third, information about the medication used is absent. Previous studies have found that the medicine used for the treatment of tic symptoms might also influence behavioral and emotional symptoms (50–52). Therefore, when we investigate the behavioral and emotional profiles in TS in future studies, we need to consider the influence of medicine, especially second-generation antipsychotics.

## Conclusion

This study explored the behavioral and emotional profiles of TS. Similar behavioral and emotional profiles were identified between TS and MDD, rather than OCD/ADHD. Aggressive behavior might be an important factor in distinguishing “pure” TS from OCD, ADHD, and MDD. More attention needs to be paid to the TP and RBB problems of the CBCL, which might have a potential influence on the functional impairments of TS.

## Data Availability Statement

The raw data supporting the conclusions of this article will be made available by the authors, without undue reservation.

## Ethics Statement

The studies involving human participants were reviewed and approved by the Ethics Committees of Beijing Children's Hospital of Capital Medical University. Written informed consent to participate in this study was provided by the participants' legal guardian/next of kin. Written informed consent was obtained from the individual(s) for the publication of any potentially identifiable images or data included in this article.

## Author Contributions

YC and YiL took the initiative. JC and YaL finished the data collection. YiL performed the data analysis and finished the draft. All authors contributed to the article and approved the submitted version.

## Funding

This work was supported by the National Natural Science Foundation of China (NSFC) under Grant No. 82001445 and 82171538, the Beijing Natural Science Foundation under Grant No. 7212035, and the Special Fund of the Pediatric Medical Coordinated Development Center of Beijing Hospitals Authority, No. XTYB201802.

## Conflict of Interest

The authors declare that the research was conducted in the absence of any commercial or financial relationships that could be construed as a potential conflict of interest.

## Publisher's Note

All claims expressed in this article are solely those of the authors and do not necessarily represent those of their affiliated organizations, or those of the publisher, the editors and the reviewers. Any product that may be evaluated in this article, or claim that may be made by its manufacturer, is not guaranteed or endorsed by the publisher.

## Supplementary Material

Supplementary Figure 1 The CBCL profile of the Young Group and Old Group.

Supplementary Table 1 The subscales of YGTSS.
