# Supplementary figures and images for: The Behavioral and Emotional Profile of Pediatric Tourette Syndrome Based on CBCL in a Chinese Sample
Source: Front Psychiatry. 2022 Feb 24;13:784753. doi: 10.3389/fpsyt.2022.784753 (PMC8907575; doi:10.3389/fpsyt.2022.784753)

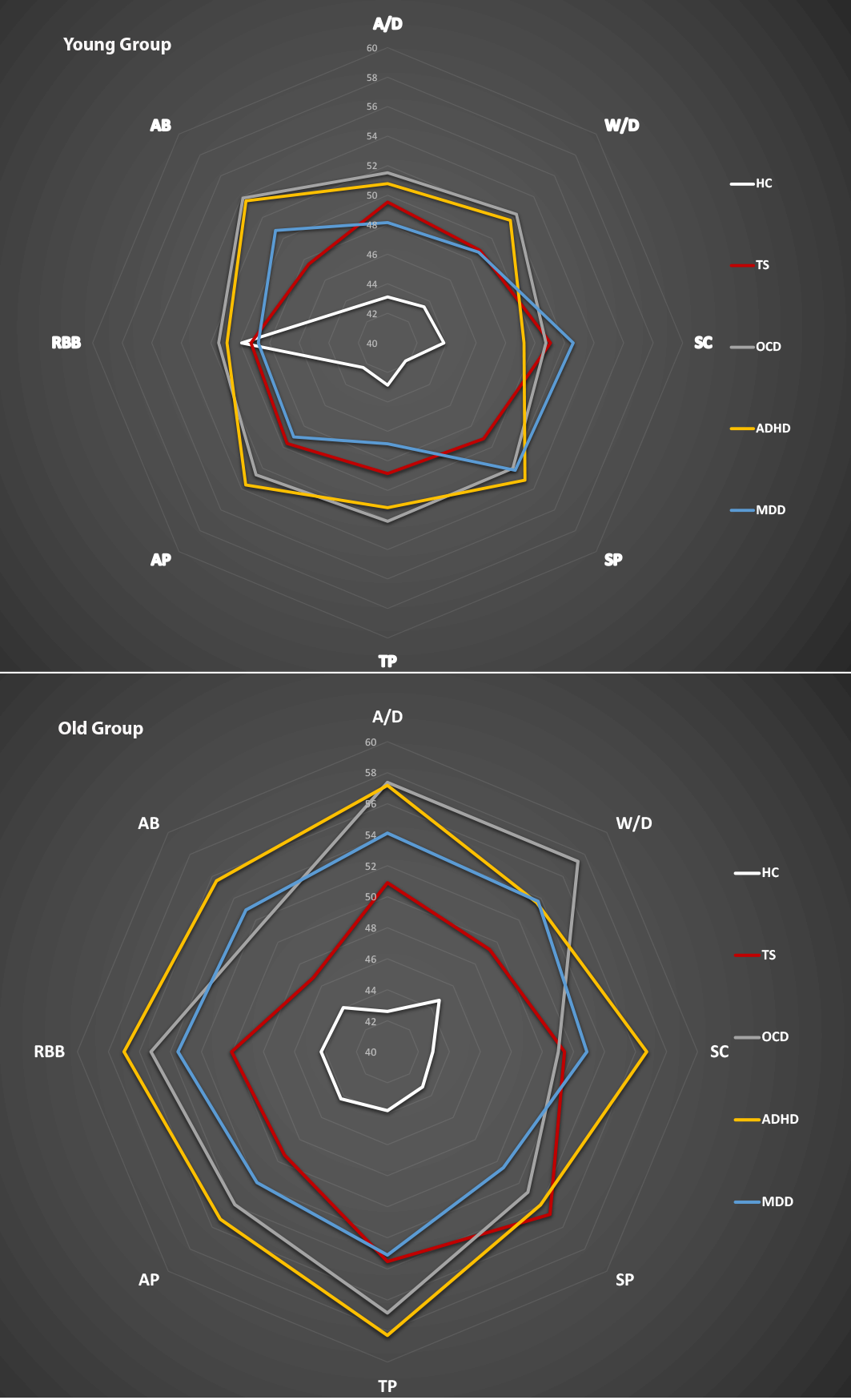

Supplement: Supplementary Figure 1 — The CBCL profile of the Young Group and Old Group. [file Figure_1.TIF]
