# Supplementary material for: The Behavioral and Emotional Profile of Pediatric Tourette Syndrome Based on CBCL in a Chinese Sample
Source: Front Psychiatry. 2022 Feb 24;13:784753. doi: 10.3389/fpsyt.2022.784753 (PMC8907575; doi:10.3389/fpsyt.2022.784753)
Supplement: Supplementary Table 1 — The subscales of YGTSS. [file Table_1.docx]

| **Subscales** | **Min** | **Max** | **Mean** | **SD** |
| --- | --- | --- | --- | --- |
| **Vocal Tic** | 5 | 19 | 10.50 | 2.38 |
| **Motor Tic** | 7 | 20 | 13.59 | 3.03 |
| **Impairment** | 10 | 30 | 13.50 | 5.58 |

**Supplementary Table 1. The subscales of YGTSS**

Note: Min, Minimum Value; Max, Maximum; SD, Standard Deviation; YGTSS, Yale Global Tic Severity Scale.
